# Acute kidney injury and ANCA positivity in a patient treated with glecaprevir/pibrentasvir: a case report

**DOI:** 10.3389/fmed.2024.1434497

**Published:** 2025-01-15

**Authors:** Lawrence Hyun Kwon, Jennifer Griffiths, Lanny DiFranza

**Affiliations:** ^1^Department of Nephrology, Westchester Medical Center Advanced Physician Services, Mid-Hudson Regional Hospital, Poughkeepsie, NY, United States; ^2^Department of Pathology, Montefiore Medical Center, Bronx, NY, United States

**Keywords:** acute kidney injury, ANCA-associated vasculitis, acute tubulointerstitial nephritis, glecaprevir/pibrentasvir, drug-induced nephrotoxicity, hepatitis C virus treatment

## Abstract

**Background:**

Glecaprevir/pibrentasvir is an effective antiviral therapy for hepatitis C virus infection and is generally regarded safe in patients with renal impairment. However, renal complications are a notable, albeit rare, concern.

**Case presentation:**

We report a case of acute kidney injury in a man in his 50s with chronic hepatitis C virus, chronic obstructive pulmonary disease, morbid obesity, a history of heroin dependence, and untreated type 2 diabetes mellitus. About four weeks into an eight-week glecaprevir/pibrentasvir regimen he developed progressive lower extremity edema, bullae, and skin ulcers with worsening renal function. His serum creatinine rose to 4.46 mg/dL and blood urea nitrogen to 44 mg/dL. ANCA serology revealed dual perinuclear and cytoplasmic positivity, though anti-proteinase 3 and anti-myeloperoxidase antibody tests were negative. Kidney biopsy revealed diffuse tubulointerstitial injury with erythrocyte casts indicative of glomerular bleeding into the distal nephrons, though without glomerular crescent formation.

**Conclusion:**

This case illustrates the potential for glecaprevir/pibrentasvir to induce acute kidney injury, acute interstitial nephritis and possibly ANCA-associated vasculitis. Recognizing these adverse renal effects is critical for making timely diagnosis and management in hepatitis C virus patients undergoing antiviral therapy.

## Introduction

Glecaprevir, a protease inhibitor, and pibrentasvir, an NS5A inhibitor, are used together for their potent antiviral effects against hepatitis C virus (HCV) infection. The glecaprevir/pibrentasvir combination has a favorable safety profile, even in patients with severe renal impairment because of its minimal renal clearance. Thus, the combination drug has become a preferred treatment for patients with advanced kidney disease, including those on hemodialysis (1), and documented adverse renal effects have been limited to isolated case reports: acute kidney injury (AKI) presenting as hepatorenal syndrome (2) and renal dysfunction in a deceased donor renal transplant recipient (3). Severe adverse effects, such as acute interstitial nephritis or drug-induced antineutrophil cytoplasmic antibody (ANCA)-associated vasculitis, evidently rarely occur with the regimen. This report presents a case of AKI in a patient receiving glecaprevir/pibrentasvir.

## Case report

A man in his 50s with a history of heroin abuse (clear of use for several years), morbid obesity, chronic obstructive pulmonary disease, and untreated type 2 diabetes mellitus presented with a two-week history of worsening lower extremity edema, bullae, and ulcers. Six months earlier, the diagnosis of HCV and liver cirrhosis was made. Approximately three months before admission, he began an eight-week course of glecaprevir and pibrentasvir (100 mg/40 mg, three tablets once daily). During the treatment, he gradually developed lower extremity edema, skin ulcers, and sensory loss. About two weeks after completing the antiviral regimen, he noted intermittent and increasingly frequent episodes of tea-colored urine and suprapubic pain. In the week leading to admission, he experienced several episodes of gross hematuria and occasional bloody stools. Three years earlier, his serum creatinine was 0.66 mg/dL and hemoglobin A1c 7.9%. About one month into his HCV therapy, his serum creatinine had increased to 0.91 mg/dL.

At the time of admission, he had completed the full eight-week glecaprevir/pibrentasvir regimen. His vital signs were stable. Physical examination revealed large, ruptured blisters and ulcers on both legs, hyperpigmentation, lipodermatosclerosis, and severe pitting edema. Arterial and venous ultrasound examinations of the legs revealed no significant stenosis or deep vein thrombosis. Renal ultrasound examination revealed enlarged kidneys, with lengths of 15 cm and 15.7 cm compared with 13 cm bilaterally six months earlier. Echocardiography revealed normal biventricular function.

Laboratory tests revealed a significant rise in blood urea nitrogen and serum creatinine, dysmorphic red blood cells in urinalysis, and elevated ANCA titers with dual perinuclear (p-ANCA) and cytoplasmic (c-ANCA) patterns, but anti-proteinase 3 (PR3) and anti-myeloperoxidase (MPO) antibody values were not elevated (Table 1). Cryoglobulin and HCV RNA tests were negative. *Streptococcus agalactiae* were cultured from urine and yeast from stool; and anti-streptolysin O titers were 400.

**Table 1:** Laboratory data summary.

Parameter	Admission	Discharge	3 months post-discharge	9 months post-discharge	Normal range
Hgb/Hct (g/dL, %)	9.9/30.9	8.2/25.6	-	12.2/37.8	13.8–17.2/40–50
Platelets (k/mm^3^)	180	107	-	89	150–450
PT/INR/aPTT (sec)	15.5/1.34/31.2	-	-	-	11–13.5/0.8–1.2/30–40
Na/K/Cl (mmol/L)	134/4.9/103	135/4.3/104	-	-	135–145/3.5–5.1/98–107
CO₂ (mmol/L)	22	20	-	-	22–29
BUN/Cr (mg/dL)	44/4.46	44/5.33	23/2.0	-	6–20/0.84–1.21
Cys C (mg/L)	-	-	-	2.32	0.6–1.0
AST/ALT/Alk Phos (U/L)	29/20/157	27/18/134	-	-	10–40/7–56/44–147
TBili (mg/dL)	0.8	0.6	-	-	0.1–1.2
TP/Alb (g/dL)	8.3/2.5	7.4/2.4	-	-	6.3–8.2/3.5–5.0
BNP (pg/mL)	826	-	7,132	-	<100
ANCA (Titer)	1:80 P/C	1:40 P/C	-	1:40 P	Negative
PR3/MPO (units)	7.85/4.93	4.85/3.09	-	6.01/2.65	<21
C3/C4 (mg/dL)	138/12	142/12	-	152/13	90–180/15–45
Cryoglobulins	Negative	-	-	-	Negative
HCV Ab	Positive	-	-	-	Negative
HCV RNA	Undetectable	-	-	-	Not detected
HBV surface Ab	Positive	-	-	-	Negative
HBV surface Ag	Negative	-	-	-	Negative

Hgb/Hct: Hemoglobin/Hematocrit; Platelets: Platelet Count; PT/INR/aPTT: Prothrombin Time /International Normalized Ratio/ Activated Partial Thromboplastin Time; Na/K/Cl: Sodium/Potassium/Chloride; CO₂: Bicarbonate; BUN/Cr: Blood Urea Nitrogen/Creatinine; Cys C: Cystatin C; AST/ALT/Alk Phos: Aspartate Aminotransferase/Alanine Aminotransferase/Alkaline Phosphatase; TBili: Total Bilirubin; TP/Alb: Total Protein/Albumin; BNP: B-type Natriuretic Peptide; ANCA: Anti-Neutrophil Cytoplasmic Antibodies; P: Perinuclear pattern; C: Cytoplasmic pattern; PR3/MPO: Proteinase 3 Antibodies/Myeloperoxidase Antibodies; C3/C4: Complement Components 3 and 4; Cryoglobulins: Serum Cryoglobulins; HCV Ab: Hepatitis C Virus Antibody; HCV RNA: Hepatitis C Virus RNA; HBV Surface Ab/Ag: Hepatitis B Virus Surface Antibody/Antigen.

**Figure 1:**
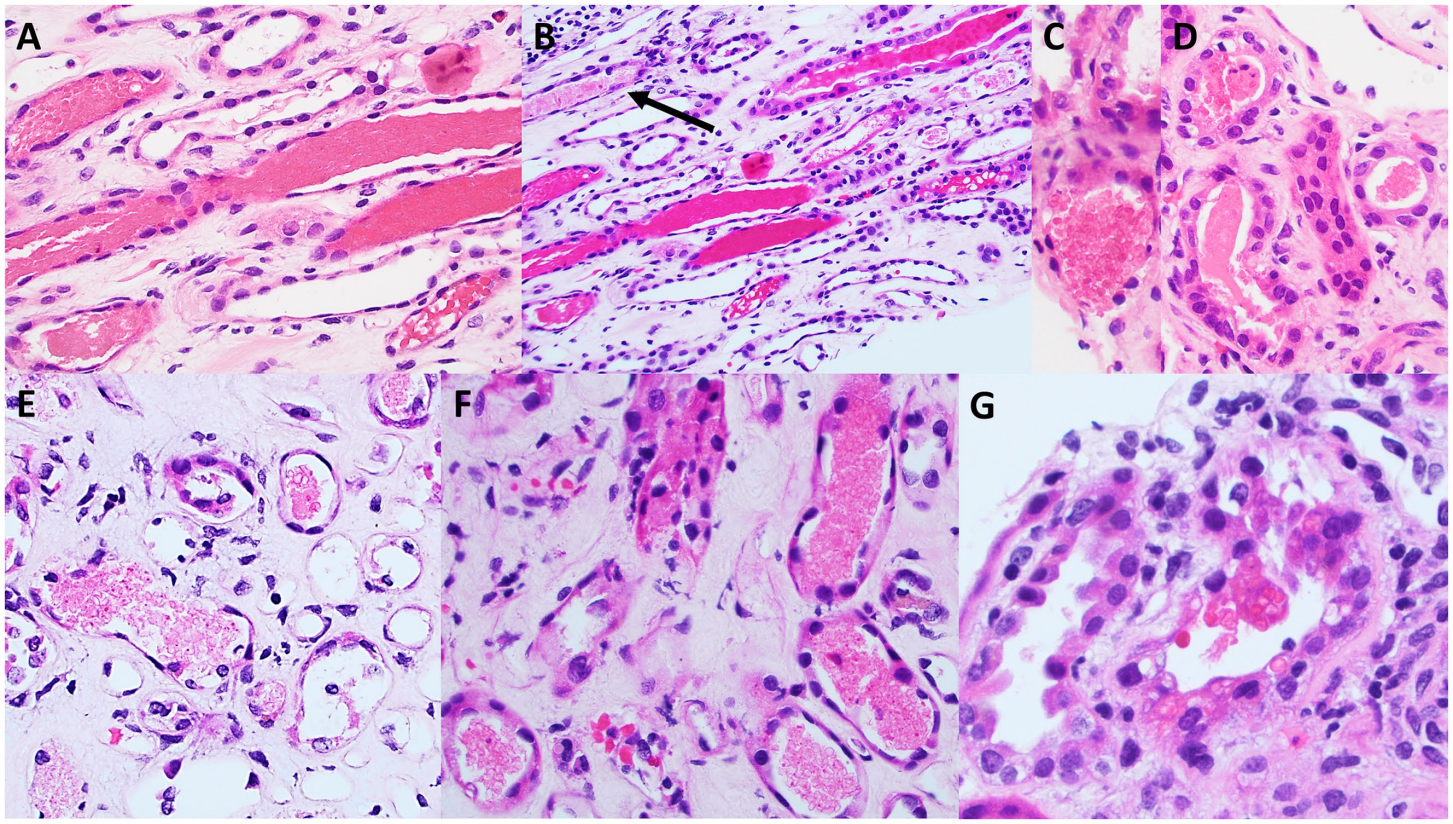
Light microscopy appearance of erythrocyte casts within the renal cortex and medulla. Scattered intratubular erythrocyte casts are present in the renal medulla **(A,B)**. Scattered granular epithelial cell casts are also present (black arrow in **B**). Erythrocyte casts are present also within the sampled cortex **(C,D)**, in addition to occasional granular epithelial cell casts (black arrow in **D**). Many of the medullary erythrocyte casts show “ghost” erythrocytes **(E,F)** that may be falsely regarded as granular epithelial cell casts when examined at low power. A rare cortical tubule shows erythrocytes incorporated into the cytoplasm of tubular epithelial cells **(G)**, providing evidence that this bleeding into tubules occurred prior to the biopsy procedure, and is not artifactual in nature [**A,B** (hematoxylin & eosin), original magnification, 40x; **C–G** (hematoxylin & eosin), original magnification, 100x].

**Figure 2:**
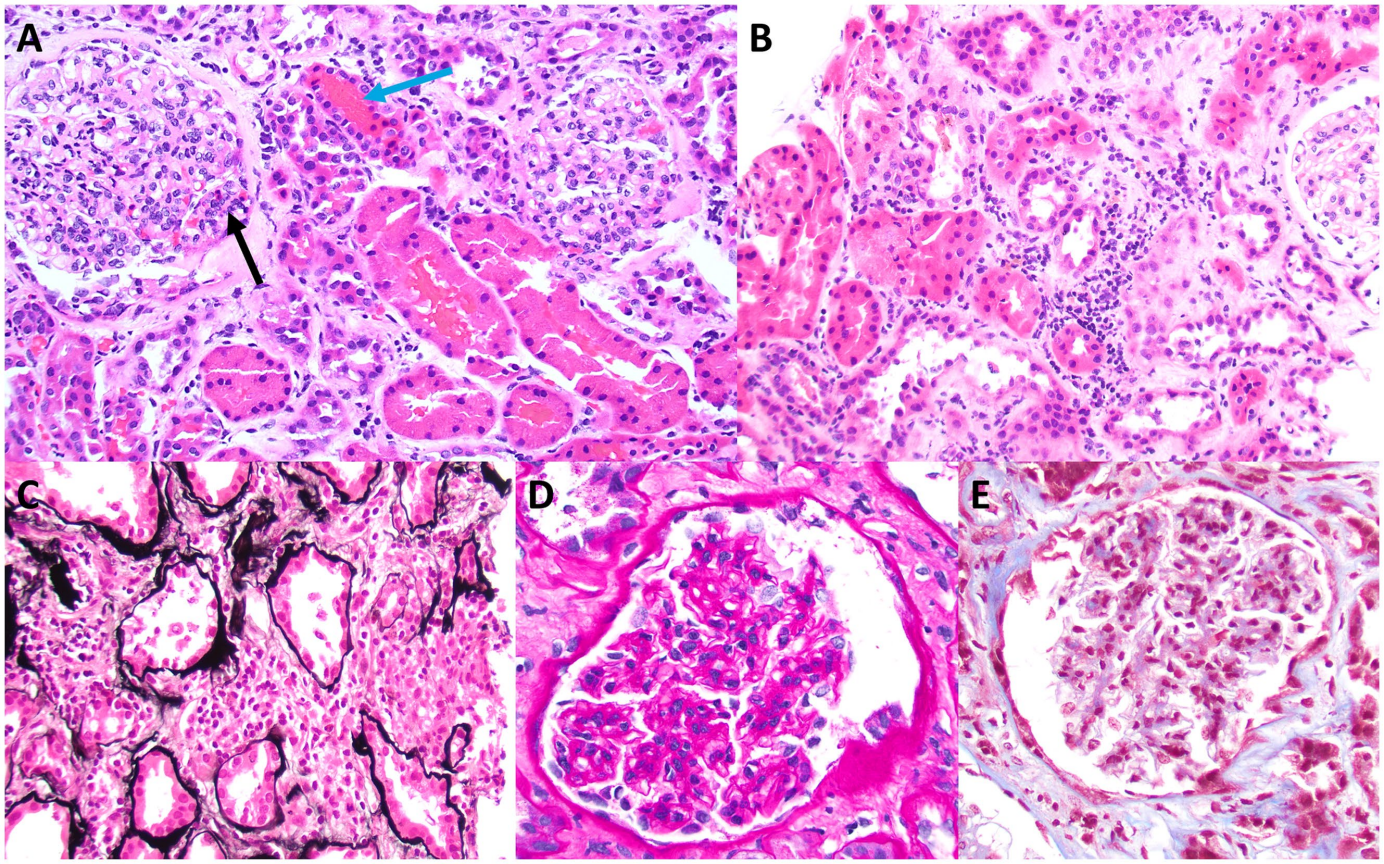
Light microscopy appearance of glomeruli and interstitial inflammation. Although no definitive endocapillary hypercellularity is present within the sampled glomeruli, rare trafficking neutrophils are present in glomerular capillaries (black arrow in **A**), a finding that is commonly seen in ANCA-related glomerular disease. There also are adjacent intratubular erythrocyte casts (blue arrow in **A**), which raise suspicion for unsampled crescentic glomerular disease **(A)**. Some areas of sampled cortex have mixed interstitial inflammation with mononuclear leukocytes, plasma cells, eosinophils, and rare neutrophils, without significant associated tubulitis, although there is evidence of acute tubular injury **(B)**. A rare focus of vaguely granulomatous inflammation by histiocytes with admixed inflammatory cells is present within the cortical interstitium **(C)**, which also raises suspicion for unsampled ANCA glomerulonephritis. Granulomatous interstitial inflammation is not an uncommon finding in cases of ANCA-associated kidney disease. Examination of sampled glomeruli reveals no definitive lesions of fibrinoid necrosis or crescent formation but demonstrates mild mesangial hypercellularity and sclerosis, with associated thickening of glomerular capillary walls, consistent with early diabetic glomerular disease **(D,E)** [**A,B** (hematoxylin & eosin), original magnification, 40x; **C** (Jones methenamine silver), original magnification, 40x; **D** (periodic acid-Schiff), original magnification, 100x; **E** (Masson trichrome), original magnification, 100x].

Skin biopsy was not obtained; however, a kidney biopsy (Figures 1, 2) was performed, with sampling of 21 glomeruli. There was no significant immunofluorescence staining with the standard panel of immunoreactants used in the evaluation of native kidney biopsies, and electron microscopy ruled out glomerular immune-complex deposition. Light microscopy revealed intratubular erythrocyte and granular epithelial cell casts within the cortex and the medulla. “Ghost” erythrocytes, which could be misinterpreted as granular casts at low magnification (Figures 1E,F), were present, along with evidence of pre-biopsy intratubular bleeding (Figure 1G). Neutrophils were present in glomerular capillaries (Figure 2A), suggesting unsampled crescentic disease, though no definitive lesions of endocapillary hypercellularity were seen. There was no arteritis. Mixed interstitial inflammation consisted of mononuclear leukocytes, plasma cells, eosinophils, and rare neutrophils, with focal granulomatous inflammation (Figure 2C). Although there were no overt signs of fibrinoid necrosis or crescent formation, early diabetic glomerular changes, including mesangial hypercellularity and capillary wall thickening, were noted (Figures 2D,E).

The patient’s management included antibiotics (initially piperacillin-tazobactam, later amoxicillin-clavulanate). Despite treatment, serum creatinine values remained elevated. Acute interstitial nephritis and ANCA-associated vasculitis, glucocorticoids were suggested, but the patient declined and preferred outpatient follow-up. At discharge, ANCA titers were again elevated (1:40), with p-ANCA and c-ANCA patterns, PR3 at 4.85 units, MPO at 3.09 units, complement levels of C3 142 mg/dL and C4 12 mg/dL.

Three months later, he was admitted for severe anasarca and a 40-lb weight gain; B-type natriuretic peptide was elevated to 7,132 pg./mL. He was treated with IV furosemide and discharged on oral furosemide. His serum creatinine was approximately 2 mg/dL, and imaging revealed pulmonary edema.

Nine months after discharge, edema and skin ulcerations had improved. Serum cystatin C was 2.32 mg/L, reflecting an eGFR of 26 mL/min.

## Discussion

We have identified only two case reports of AKI after glecaprevir/pibrentasvir therapy (2, 3). In one of the patients (2), who had hepatorenal syndrome, renal function recovered after discontinuation of the drug. In the other (3), partial recovery occurred without a renal biopsy to characterize the kidney injury. In general, glecaprevir/pibrentasvir is well tolerated; the major adverse effects are nausea and diarrhea (1). Glecaprevir, an NS3/4A protease inhibitor, prevents viral replication by blocking the cleavage of the HCV polyprotein. Pibrentasvir, an NS5A inhibitor, interferes with HCV RNA replication and virion assembly. The combination drug offers robust antiviral effects across all HCV genotypes and is approved for treating chronic HCV in patients aged 12 and older (1).

In this case, cryoglobulin tests were negative, ruling out cryoglobulinemia, a common HCV-related cause of renal dysfunction (4). We propose two possibilities two to explain the kidney injury: drug-induced (1) acute interstitial nephritis and (2) ANCA-associated vasculitis. Red blood cell (RBC) casts can be present in acute interstitial nephritis, and perhaps AKI was due to acute tubulointerstitial injury from intratubular RBC obstruction and phagocytosis by tubular cells (5). This mechanism, involving reversible tubular damage, may account for the eventual partial recovery in renal function. The presence of RBC casts in acute interstitial nephritis can occur when inflammation disrupts interstitial vessels, allowing RBCs to leak into the interstitium and enter tubule lumens through breaks in the basement membrane. Tubular injury associated with intratubular RBCs and RBC casts has been documented (5).

Drug-induced ANCA vasculitis is also possible. While ANCA positivity can occur in HCV infections without associated vasculitis—often with titers rising in response to viremia due to an immune reaction to the virus (6)—active HCV was ruled out in this case, as HCV RNA was negative following glecaprevir/pibrentasvir treatment, indicating viral clearance. Drug-induced vasculitis predominantly affects the skin, presenting with necrotic lesions, hemorrhagic bullae, and ulceration similar to those seen in this patient (7). Unfortunately, no skin biopsy was obtained to confirm the presence of vasculitis.

Other HCV antiviral treatments have also been associated with ANCA vasculitis. For instance, sofosbuvir and ribavirin therapy has led to ANCA-associated glomerulonephritis (8), presumably through the conversion of drugs into cytotoxic metabolites that prime neutrophils to display MPO or PR3 antigens on their cell membranes. When ANCA binds to these antigens, it can result in excessive neutrophil activation, cytokine release, production of reactive oxygen species, and vascular damage (9). However, MPO and PR3 levels were within normal limits in this patient, and atypical ANCAs—autoantibodies targeting other neutrophil components, such as human leukocyte elastase, cathepsin G, lactoferrin, and bactericidal/permeability-increasing protein—were not assessed.

In some cases, ANCA-associated nephritis may present without crescents and with atypical features, such as peritubular capillaritis and granulomatous interstitial inflammation, as seen in certain cases of ANCA nephritis without crescent formation (10).

If this patient did not have ANCA drug-induced vasculitis, what explains the positive ANCA indirect immunofluorescence results? First, inherent challenges in ANCA testing must be considered. ANCA testing primarily uses indirect immunofluorescence to detect two main patterns: cytoplasmic and perinuclear, and the interpretation of the results is subjective, with the lack of standardized protocols across clinical laboratories causing variability in sensitivity and specificity (11, 12). False positives or false negatives can occur, especially with low ANCA titers (11, 12). Cross-reactivity with autoantibodies such as anti-nuclear antibody, anti-Jo-1, or anti-ribosomal nucleoprotein can further complicate interpretation, as these can produce fluorescence patterns resembling p-ANCA and c-ANCA (11, 12).

ANCA elevations also can occur for reasons other than systemic vasculitis, as in connective tissue diseases, inflammatory bowel disorders, autoimmune liver disease, malignancies, myelodysplastic syndromes, and even diabetes mellitus (13). ANCA titers may rise during infections, underscoring the importance of excluding infectious causes before initiating immunosuppressive therapy. In such cases, transient ANCA elevation may result from molecular mimicry rather than active vasculitis (13).

Although the anti-streptolysin O titer was positive and the urine culture grew *Streptococcus agalactiae*, these findings do not necessarily indicate infectious glomerulonephritis. Infectious glomerulonephritis, particularly post-streptococcal, typically presents with immune complex deposits within the glomeruli, often appearing as subepithelial “humps” on electron microscopy, along with mesangial or endocapillary proliferation on light microscopy (14). In this patient’s renal biopsy, these characteristic features were absent, making an infectious cause less likely. However, streptococcal infections have also been implicated in interstitial nephritis (15), which warrants consideration in this context.

It is important to note that AKI can contribute to acute heart failure through various mechanisms, including salt and water retention, activation of the renin-angiotensin and sympathetic nervous systems, increased cardiac afterload, and inflammatory responses. Three months post-discharge, this patient exhibited signs of heart failure, potentially linked to the AKI episode. This aligns with a study of 31,245 AKI patients matched with 146,941 non-AKI patients, which found that AKI was associated with a 44% increased risk of developing heart failure within one year (16).

## Conclusion

This case highlights a unique instance of acute kidney injury potentially linked to the hepatitis C treatment regimen of glecaprevir/pibrentasvir. Although the patient was ANCA-positive with cutaneous features often seen in ANCA vasculitis, the absence of elevated MPO and PR3 antibodies, lack of glomerular crescent formation, and presence of tubulointerstitial injury suggest that the renal manifestations may have resulted from drug-induced acute interstitial nephritis rather than classic ANCA-associated vasculitis. Further research is needed to clarify the renal safety profile of the glecaprevir/pibrentasvir regimen, particularly in individuals with pre-existing risk factors.

## Data Availability

The original contributions presented in the study are included in the article/supplementary material, further inquiries can be directed to the corresponding author.
